# Bottlenecks in the Coverage of the Anemia Mukt Bharat Programme in Rural Jharkhand and the Way Forward: A Qualitative Research

**DOI:** 10.7759/cureus.91087

**Published:** 2025-08-27

**Authors:** Aishwarya Bhushan, Asim A Minj, Anit Kujur, Vidya Sagar, Alka Rashmi Nag

**Affiliations:** 1 Community Medicine, Rajendra Institute of Medical Sciences, Ranchi, IND; 2 Surgery, Rajendra Institute of Medical Sciences, Ranchi, IND; 3 Preventive Medicine, Rajendra Institute of Medical Sciences, Ranchi, IND; 4 Anatomy, Rajendra Institute of Medical Sciences, Ranchi, IND

**Keywords:** anemia mukt bharat, bottlenecks, jharkhand, qualitative, rural

## Abstract

Background

Exorbitant prevalence of anaemia is a major public health perturbation in India. The objectives of this study are to explore the reasons behind the non-acceptability of the interventions among pregnant and lactating females, provided under the Anemia Mukt Bharat (AMB) programme, including the beneficiaries' perceptions, systemic issues, and cultural factors, and to describe the challenges faced by service providers in delivering the services.

Methods

A qualitative study was conducted to explore the reasons behind the non-acceptability of the interventions provided under the AMB programme, from July 2023 to June 2024, in the Ormanjhi block of Ranchi district in Jharkhand. An in-depth Interview was performed with 95 pregnant and lactating females above 18 years of age, recruited by stratified non-random purposive sampling. Two focus group discussions were done separately with auxiliary nurse midwives (ANMs) and accredited social health activists (ASHAs) of the area, and two key informant interviews were done with the medical officer in charge (MOIC) and child development project officer (CDPO) of the block.

Results

Thematic analysis revealed four major themes with 10 sub-themes, reflecting the various behavioural, infrastructural, and systemic impedances to the effective implementation of the program. These primarily included irregular supply and frequent stockouts of the supplements at the healthcare facility, poor compliance due to side effects associated with the supplements, lack of awareness among the beneficiaries, lack of availability of the information, education, and communication (IEC) materials, existing cultural beliefs and barriers, and the educational qualifications of the beneficiaries. The main challenges faced by the service providers under the programme were a shortage of manpower and increased workload, frequent stockouts of the supplements and IEC materials, inadequate periodic training provided to the ground-level workers on the functioning mechanism of the programme, lack of advocacy from local leaders, and a meagre pay scale of frontline workers.

Conclusion

The present study reveals critical insights into the programme's reach and effectiveness, indicating a substantial gap between the programme's objectives and its actual implementation on the ground. Despite the programme's well-intentioned efforts to combat anaemia, the coverage remains inconsistent and limited. Several factors contribute to this shortfall.

## Introduction

Exorbitant prevalence of anaemia is a crucial public health perturbation in India that seeks redressal through multifarious prospects and through numerous aligned endeavours and attempts. The first-ever national anaemia control programme was started in 1970, known as the National Nutritional Anemia Prophylaxis Programme (NNAPP) [1,2]. It has evolved over the years to the currently ongoing Intensified National Iron Plus Initiative (I-NIPI), which is popularly known as Anemia Mukt Bharat (AMB) [3,4].

AMB is one of the 15 nutrient interventions delivered through the National Health Mission (NHM) working upon the 6 x 6 x 6 strategy to combat anaemia [5,6,7]. It has six beneficiary groups, six interventions, and six institutional mechanisms working behind it to reduce the prevalence of anaemia by 3% per year [8,9]. The six beneficiaries roped into the programme include children (six to 59 months), adolescent males and females (10-19 years), women of reproductive age group (15-49 years), and pregnant and lactating females (zero to six months). Six key interventions provided include (a) prophylactic iron and folic acid (IFA) supplementation; (b) biannual deworming; (c) intensified year-round behaviour change communication (BCC); (d) digital testing of anaemia; (e) IFA fortification of foods in government-run health programmes; and (f) addressing non-nutritional causes of anaemia. Six institutional mechanisms under this ambitious initiative comprise intra-ministerial coordination, strengthening supply chain and logistics, the National Centre of Excellence and Advanced Research on Anemia Control, the National AMB unit, convergence with other ministries, and the AMB dashboard and digital portal that is a one-stop shop for anaemia [8]. In addition to I-NIPI, there are other programmes as well, which address the non-nutritional causes of anemia, such as the National Deworming Day (NDD) for deworming, [10] National Vector Borne Disease Control Programme (NVBDCP) for malaria and special actions and efforts to reach out to the marginalised populations affected with haemoglobinopathies and sickle cell anemia, and lastly the National Programme for Prevention and Control of Fluorosis.

Despite the strong government commitments, the magnitude of this problem still remains alarmingly high, especially when it comes to pregnant and lactating females. According to the data in the National Family Health Survey (NFHS-5), 45.9% of pregnant women in Ranchi are anaemic, and only 20% consumed IFA for 180 days during pregnancy [11].

National and global lessons learnt so far clearly indicate that if dedicated and targeted efforts are made for anaemia reduction through the highest achievable political commitment, target-setting approach across the age groups, strengthening the coverage of the programme, addressing failures in procurement and the supply chain management issues with a robust monitoring framework and responsive review systems. With intensive BCC campaigns, with special focus on the particularly and potentially vulnerable geographies, it is rather possible to achieve the desired goals.

This study was done with an objective to explore the reasons behind the non-acceptability of the interventions among pregnant and lactating females, provided under the AMB programme, including the beneficiaries' perceptions, systemic issues, and cultural factors, and to describe the challenges faced by the service providers in delivering the services.

## Materials and methods

A qualitative study consisting of in-depth interviews (IDIs) with the beneficiaries, focus group discussions (FGDs), and key informant interviews (KIIs) with service providers was conducted after the Institutional Ethical Committee approval (13/IEC/RIMS dated 2/2/23). This study was conducted over a 12-month duration, starting from July 2023 to June 2024, in the Ormanjhi Block of Ranchi District, Jharkhand.

IDIs provided the beneficiaries' perceptions on non-acceptance of services, whereas FGDs and KIIs provided the challenges faced by the service providers and their perceptions on the non-acceptance of services among the beneficiaries. Incorporation of these three methods provided a complete picture of the existing ground reality of program implementation.

An interview guide consisting of open-ended questions was pre-tested with three pregnant and three lactating females for its lucidity and content and was finalised after necessary modifications. A small number (six) of participants served the purpose of checking the clarity, flow, and contextual appropriateness of the tool rather than to achieve data saturation. Three participants per group were adequate to identify major comprehension issues, redundancies, and cultural sensitivities. This number strikes a balance between feasibility and ensuring that more than one perspective within each beneficiary category is captured.

Stratified non-random purposive sampling was done to recruit participants from five different sub-centres of Ormanjhi block for IDI after receiving their written informed consent. A line-list of all the pregnant and lactating females was obtained from all the Anganwadi centres under each sub-centre, and then the females not availing the services were approached for the IDI. This included 95 pregnant and lactating females above 18 years of age and residents of Ormanjhi block who were not consuming the supplements provided by the government. Females who refused to give their consent for the same were excluded. No females with medical contraindication to the supplements were encountered. Theoretical saturation of the data guided the final sample size. House visits were done for the collection of data. Each interview lasted for an average duration of 25 to 30 minutes. All the interviews were conducted by the same pair of researchers to maintain uniformity.

Two KIIs with the MOIC and CDPO were conducted at their respective offices, after taking prior permission, and two FGDs with the ANMs and ASHAs separately were conducted to get an in-depth knowledge of challenges faced by service providers of the programme. An FGD was conducted using the FGD Guide at the Community Health Centre, Dunde, Ormanjhi, on the days of scheduled monthly cluster meetings after taking permission from the MOIC. One FGD was done with eight Sahiyas/ASHAs and one FGD with nine ANMs, each with around 40 minutes' duration.

Interviews were carried out in the local language, in a quiet and open area, audio-recorded and transcribed verbatim. Interview data was analysed thematically. All the audio recordings were kept in a password-protected drive folder.

A multi-method qualitative analysis approach was deployed to ascertain a comprehensive understanding of the bottlenecks in the program implementation in rural Jharkhand. The analysis included inductive thematic analysis, wherein the researchers read the transcripts multiple times and noted the initial ideas. Thereafter, open coding was conducted manually by a single researcher to ensure consistency in interpretation. Peer debriefing was done throughout to discuss the codes with the colleagues and to capture relevant concepts such as "fear of side effects" and "frequent stockouts of IFA tablets". Member checking was not done as it was practically not possible to trace back the interviewees. Coded data were grouped into potential themes that reflected patterned responses or shared meanings. Themes were reviewed to ensure that they accurately represented the coded data and aligned with the full dataset. Any overlapping or ambiguous themes were refined. Each theme was named to reflect its core meaning. Sub-themes were identified to reflect nuanced findings. Themes were further illustrated using representative quotes from participants [12].

Data from the IDIs, FGDs, and KIIs were first analysed separately to identify preliminary themes. Themes were then compared across methods to identify points of convergence, complementarity, and divergence. A thematic matrix was developed to integrate the findings, allowing triangulation of all the perspectives. Final themes were presented in an integrated manner, with illustrative quotes drawn from all three data sources.

Themes from the beneficiaries' and providers' interviews were analysed separately to capture their distinct perspectives. Subsequently, these themes were compared and integrated to identify convergence, complementarities, and discrepancies, thereby ensuring a comprehensive understanding of the issue.

A deductive framework analysis was further incorporated to map the coded data of identified barriers against the four strategic pillars of the programme, serving as the conceptual framework to align the key themes with programme components [13].

Content analysis was employed to quantify the frequency of key barriers and beliefs emerging from qualitative data, with an aim to identify the most recurrent themes across participant narratives. The unit of analysis was each individual interview or the FGD session, and the code was counted once per transcript to avoid overrepresentation, with a total n = 99 [14]. This triangulated approach ensured the emergence of inductive themes along with systematic alignment with the AMB programme structure and quantification of the most frequently cited barriers and beliefs. The findings were illustrated with the aid of an image created by the author using Microsoft PowerPoint (Microsoft Corp., USA).

## Results

The present study revealed that only 16 (16.8%) of the 95 subjects had heard about the AMB programme. Out of them, three were told about it by Sahiya/ASHA during the home visit. Eleven were informed about it at the healthcare facility during their ANC visit, and two were told about the programme at their schools.

**Figure 1:**
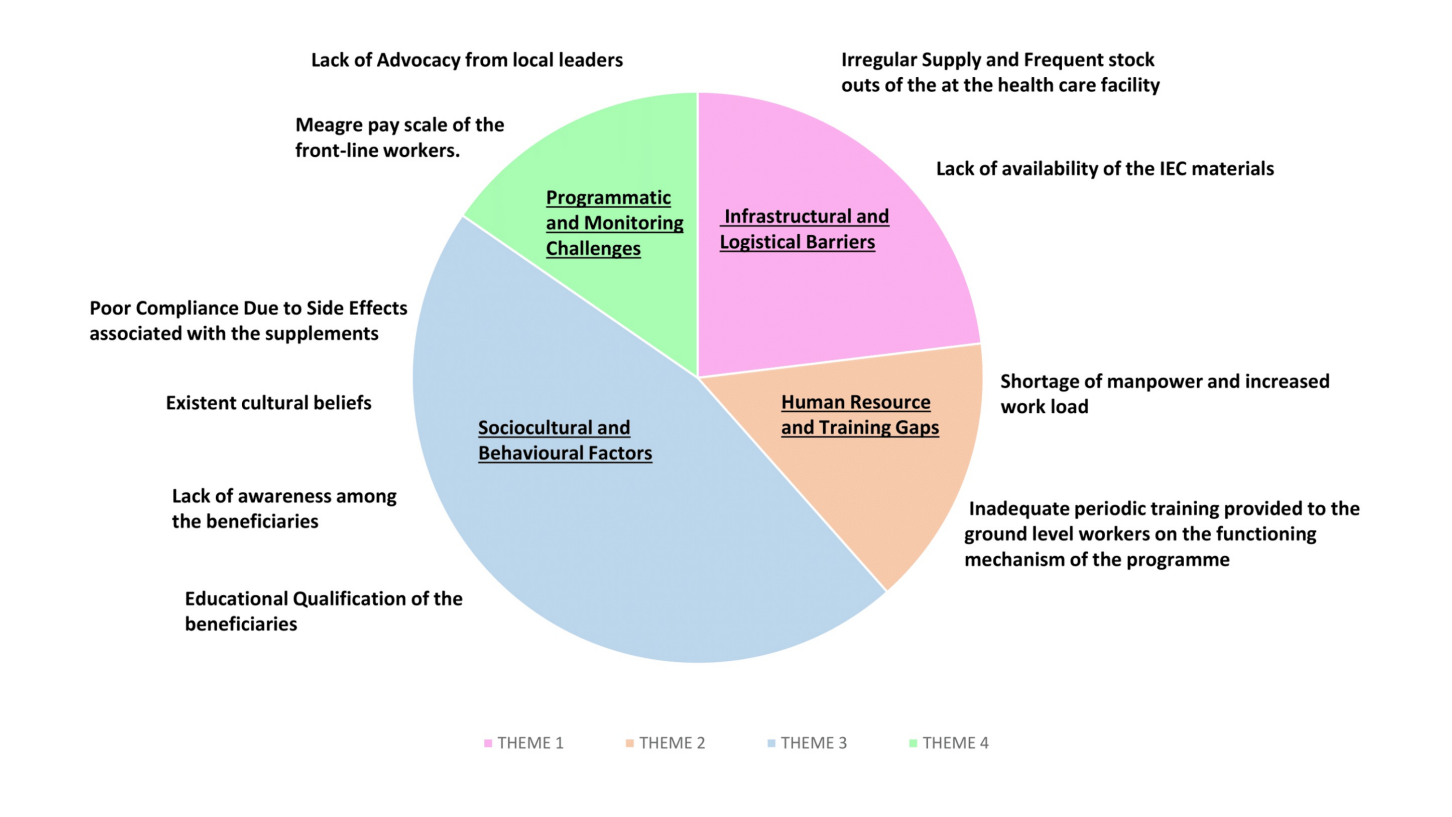
Diagrammatic representation of various themes along with their respective sub-themes

Thematic analysis revealed four major themes with 10 sub-themes, reflecting the various behavioural, infrastructural, and systemic impedances to the effective implementation of the program (Figure 1).

Theme 1: infrastructural and logistical barriers

Sub-theme 1.1: Irregular Supply and Frequent Stockouts of the Supplements at the Healthcare Facility

All the healthcare workers from all the centres informed that the supplements were out of stock for the entire last year, for the complete block, and they received them some two to three months back.

“IFA tablets were out of stock for the past few months in the entire Ormanjhi block. We received the stocks two months back. There were many females who did not buy the supplements as they are completely reliant on government supply” - (ANM)

Sub-theme 1.2: Lack of Availability of the IEC Materials

Many ANMs informed that they do not get ample IEC materials at their centre, making it difficult for them to counsel the beneficiaries. ANMs from only two subcentres reported receiving the IEC materials.

“Since my centre lies in a remote location, I don’t even get the posters and the pamphlets of the program.” - (ANM)

“Earlier, we used to get some videos to show to the beneficiaries, which they liked and enjoyed. I have seen females listening eagerly to it and following the advice as well. But now there are no such videos available to us, and it is very difficult to make them understand by speaking” - (ASHA)

Theme 2: human resource and training gaps

Sub-theme 2.1: Shortage of Manpower and Increased Work Load

The healthcare workers at all levels experience a shortage of manpower. ASHAs complained that since they have to fill out numerous registers altogether, they do not get ample time for field visits. ANMs said that since many of the in-service ANMs have retired and no new recruitment has been done, they have to cover all the Village Health and Nutrition Day (VHND) sessions of their area, and they find it difficult to vaccinate all the children in one session. CDPO said that there are only two lady supervisors instead of five in the block, and this makes the implementation and monitoring task for her quite difficult.

“We are made to fill 50 types of registers. What is the need for register entry again if we are already doing it online” - (ASHA)

“I work more than the ANM in my area. She only knows how to give injections. I can also do that if training is provided to me” - (ASHA)

Sub-theme 2.2: Inadequate Periodic Training Provided to the Ground-Level Workers on the Functioning Mechanism of the Programme

Some health workers lacked adequate training on updated AMB guidelines, dosage, and follow-up schedules.

ANMs and ASHAs informed that they have been provided training only once regarding the programme, and before that, they were just working on verbal orders. They admitted that they rarely receive any training for a program in particular. The MOIC said that it was difficult for her to provide training to all 165 ASHAs at a time, so she divided them into small groups for this purpose.

“We only received a one-day training regarding this programme some two months back. Before that, we were working on verbal orders from above.” - (ASHA)

Theme 3: sociocultural and behavioural factors

Sub-theme 3.1: Poor Compliance Due to Side Effects Associated With the Supplements

Due to various side effects associated with the supplements, like black stool, nausea, vomiting, and abdominal pain, pregnant females usually stop taking them any further.

“There are a few females in my area who have stopped taking the tablets after experiencing side effects like black stool, nausea and vomiting. I try very hard to convince them that these side effects would go away with time, but still they refuse to eat due to the fear that this would harm their unborn child” - (ASHA)

Sub-theme 3.2: Existing Cultural Beliefs and Barriers

Taking IFA supplements would increase the size of the baby that they will eventually have to undergo caesarean section for delivery, and these supplements would reduce the breast milk output. Cultural beliefs and barriers were present and widespread, irrespective of religion, education, or sociodemographic distribution.

“My sister-in-law has told me that if I eat these supplements, the baby will grow in size and eventually I will have to undergo caesarean section” - (pregnant female)

“These supplements will reduce my breast milk output, and my baby would starve” - (lactating female)

“Rice, pulses, and soya bari provided earlier in the ration were better. At least, they consumed them. The new Ration that is given to them has a lot of salt so they feed it to the cattle” -(ASHA)

“Women these days give more importance to doctors. They think that ANM does not know anything, so why should I listen to her? She gets paid for speaking all this.” - (ANM)

Sub-theme 3.3: Lack of Awareness Among the Beneficiaries

Inadequate knowledge among the beneficiaries regarding the importance of these supplements and the availability of free supplements.

“My mother-in-law never consumed any such medicine, but all her children were born through normal delivery” - (pregnant female)

Sub-theme 3.4: Educational Qualification of the Beneficiaries

ASHAs and ANMs of two sub-centres reported that they find it particularly difficult to ensure compliance with IFA supplementation when it comes to educated females because they don’t find them competent enough to give any medical advice. So, the females do not listen to them.

 “Lack of education and awareness in the community makes it difficult to implement any new strategy. They question and revolt against small things, even.” -CDPO

“Poor people of my area are more aware and compliant. There are two rich families, and they never come to the centre upon being called. Even on repeated telephonic reminders, she says that she is out of station” -(ASHA)

Theme 4: programmatic and monitoring challenges

Sub-theme 4.1: Lack of Advocacy From Local Leaders

The healthcare providers complained that due to the non-participation of local leaders and community representatives in this work, it is very difficult for them to convince the women about the advantages of the programme. This issue was raised by the medical officer in respect to the local leaders and the private school administration. The same was also reported by ANMs of three subcentres.

“The local leader of my area refused to address the beneficiaries of the programme about the benefits, saying that he does not get paid for it.” - (MO)

Sub-theme 4.2: Meagre Pay Scale of the Frontline Workers

Particularly, the ASHAs complained that they get only rupees 2000 a month, which is quite insufficient. They have to spend more than this amount, particularly on transportation. They felt that they work much more than ANMs, but still the difference in the money they receive is substantial, and because of this, they do not find themselves motivated enough. This expression was in terms of broader concerns pertaining to ASHA remuneration and not the AMB program in particular, but it does impact the proactive participation on their part in all the activities performed by them.

“I find it shameful that in return for the service that I provide to the community, I am paid only 2000 a month. If someone asks, I lie that I get 10,000 in a month.” - (ASHA)

Table 1 shows the incorporation of the extracted sub-themes with the operational framework of the programme along with its quantitative representation.

**Table 1:** Incorporation of the extracted sub-themes with the operational framework of the programme along with its quantitative representation

S. No.	Pillars of the programme	Themes/barriers	Frequency of mention
1	Prophylactic iron and folic acid supplementation	Irregular supply and frequent stockouts of the supplements at the healthcare facility	79/99 interviews
		Poor compliance due to side effects associated with the supplements	56/99
2	Biannual deworming	Lack of awareness among the beneficiaries	43/99
3	Intensified behaviour change communication	Lack of availability of the IEC materials	39/99
		Existing cultural beliefs and barriers	67/99
		Educational qualification of the beneficiaries	24/99
		Lack of advocacy from local leaders	10/99
4	Testing and treatment of anaemia	Shortage of manpower and increased workload	5/99
		Inadequate periodic training provided to ground-level workers	4/99
		Meagre pay scale of frontline workers	5/99

## Discussion

The present study revealed that only 16 (16.8%) among the 95 subjects had heard about the AMB programme, similar to the findings in the study done by Reshmi et al. in rural areas, who also documented that only 13.3% had good knowledge of the AMB strategy [15].

Wendt AS et al. [16] found that in total, 44% of auxiliary nurse midwives lacked IFA supplies. District-to-district variations in supply chain procedures and stock levels were substantial. Specific obstacles affecting IFA forecasts, purchase, storage, and disposal, as well as a shortage of staff and limited training opportunities for important supply chain participants, were identified by qualitative data. One of the main obstacles to the IFA supplementation program is inadequate IFA supply, which varies greatly from district to district. To strengthen the IFA supply chain in Bihar, it will be essential to make improvements at all levels of infrastructure, procedures, and efficient monitoring. These findings are in complete parallelism with the findings reported in our study.

Sedlander E et al. [17] in their study found that although participants were aware that supplements can prevent anaemia on an individual basis, they misjudged the danger and prevalence of anaemia in their society. According to the participants, using excessive amounts of iron supplements during pregnancy would "make your baby big," resulting in a difficult delivery and an expensive caesarean. Relationship-wise, husbands were more accepting of their wives taking frequent iron supplements throughout pregnancy than mothers-in-law. According to the participants, only adolescents and pregnant women in the neighbourhood use iron supplements; non-pregnant women are completely ignored. Another upstream obstacle that prevents non-pregnant women from prioritising their health in order to receive iron supplements is unequal gender norms. Frontline health workers just give iron supplements to expectant mothers and do not monitor adherence at the policy level. The findings of this study offer considerable similarity to the barriers identified in our study, further strengthening the widespread presence of such beliefs.

Joe W et al. [18] in 2022 stated that IFA supplementation coverage has grown for all recipient groups between 2017-2018 and 2019-2020 following the implementation of the AMB strategy. When comparing target groups serviced by a multi-departmental convergence mechanism to those served by the health department alone, coverage was comparatively low. However, there are not any significant gender differences in how school-age boys and girls are covered by IFA supplementation. The majority of the coverage variances can be attributed to state-specific variations. There is evidence linking enhanced coverage across beneficiary categories to training and sensitisation seminars for state and district authorities. The coverage trends reported in the current study slightly differ from the reported national trends. This could be attributed to the fact that the beneficiaries covered in our study were only the pregnant and lactating females.

Ahmad K et al. [19] in 2023 stated that the evaluation of the AMB program revealed several important problems, including the absence of an integrated MIS, a fixed distribution schedule, a lack of inventory management strategies, a lack of standardised forecasting procedures, and insufficient availability of transport vehicles. The congruity reported between the studies highlights the presence of systemic barriers that seek steady redressal, in addition to the location-specific weaknesses.

In order to explore potential solutions for enhancing adherence and behaviours to prevent and cure anaemia among pregnant and breastfeeding Indian women, a formative research study was conducted by Williams PA et al. [20] to examine social and cultural aspects surrounding the provision and adherence of IFA supplements. The findings showed that while the majority of the women were aware of anaemia, they were unaware of its gravity and repercussions. IFA supplements were given to all women (mostly for free), but many did not follow through due to side effects, a lack of knowledge from medical professionals about the causes, severity, and remedies of anaemia, and a lack of social support. Although women lacked control over resources like the availability of appropriate food, they were most confident in their own ability to produce and consume better foods to treat anaemia. These findings are in direct congruence with the conclusions of our study, further reinforcing the consistency of findings from different locations.

The limitations of the study included the inclusion of only two out of the six beneficiaries and four out of the six interventions of the programme. This considerably limits the transferability of the findings to the entire program, as the experiences of other beneficiary groups and challenges related to the other interventions may differ. Further, Intervention overlap due to other existing health programmes could confound the results, making it difficult to isolate the specific impact of the programme. Contextually rich Qualitative data might not adequately represent the magnitude of each barrier across the wider population.

## Conclusions

The present study reveals critical insights into the programme's reach and effectiveness, indicating a substantial gap between the programme's objectives and its actual implementation on the ground. Infrastructural and logistical barriers, such as irregular supply, disrupt the continuity of services. Human resource shortage and training gaps further weaken the program delivery as overburdened staff and insufficiently trained workers struggle to ensure consistent counselling, distribution, and follow-up. At the community level, sociocultural beliefs and behavioural factors, including misconceptions about IFA supplementation, reduce the acceptance and compliance. Finally, the programmatic and monitoring challenges limit accountability and responsiveness.

Despite the programme's well-intentioned efforts to combat anaemia, the coverage remains inconsistent and limited. Several factors contribute to this shortfall, including inadequate awareness among the target population, insufficient supply and distribution of iron and folic acid supplements, and a lack of regular monitoring and follow-up by healthcare providers. Socioeconomic barriers, such as limited access to healthcare facilities and education, further exacerbate the issue.

To enhance the programme's impact, it is imperative to address these challenges through targeted interventions. Strengthening the supply chain management by ensuring buffer stocks at block and subcentre levels is considerable. Timely recruitment and redistribution of human resources would reduce the workload. Periodic refresher training on AMB protocols would ensure effective implementation. Intensified community-based IEC/BCC campaigns using culturally appropriate messages involving community influencers (PRI members and SHG leaders) would promote family-level counselling to enhance adherence. While the AMB programme has the potential to significantly reduce anaemia among pregnant and lactating women in Ormanjhi, concerted efforts are required to bridge the existing gaps and achieve its goals. This study underscores the need for a multifaceted approach, involving stakeholders (healthcare providers, local leaders, supply chain managers) at all levels, to ensure that no woman is left behind in the fight against anaemia and further highlights the need for further research involving other AMB beneficiaries to achieve a wider picture of the existing bottlenecks.
